# Beyond transcription factors: more regulatory layers affecting soybean gene expression under abiotic stress

**DOI:** 10.1590/1678-4685-GMB-2022-0166

**Published:** 2023-01-23

**Authors:** Isabel Cristina Cadavid, Natalia Balbinott, Rogerio Margis

**Affiliations:** 1 Universidade Federal do Rio Grande do Sul, Centro de Biotecnologia, Programa de Pós-graduação em Biologia Celular e Molecular (PPGBCM), Porto Alegre, Brazil.; 2 Universidade Federal do Rio Grande do Sul, Departamento de Genética, Programa de Pós-graduação em Genética e Biologia Molecular (PPGBM), Porto Alegre, Brazil.; 3 Universidade Federal do Rio Grande do Sul, Departamento de Biofisica, Porto Alegre, Brazil.

**Keywords:** Epigenetics, abiotic stress, histone methylation and acetylation, DNA methylation, circular RNA

## Abstract

Abiotic stresses such as nutritional imbalance, salt, light intensity, and high and low temperatures negatively affect plant growth and development. Through the course of evolution, plants developed multiple mechanisms to cope with environmental variations, such as physiological, morphological, and molecular adaptations. Epigenetic regulation, transcription factor activity, and post-transcriptional regulation operated by RNA molecules are mechanisms associated with gene expression regulation under stress. Epigenetic regulation, including histone and DNA covalent modifications, triggers chromatin remodeling and changes the accessibility of transcription machinery leading to alterations in gene activity and plant homeostasis responses. Soybean is a legume widely produced and whose productivity is deeply affected by abiotic stresses. Many studies explored how soybean faces stress to identify key elements and improve productivity through breeding and genetic engineering. This review summarizes recent progress in soybean gene expression regulation through epigenetic modifications and circRNAs pathways, and points out the knowledge gaps that are important to study by the scientific community. It focuses on epigenetic factors participating in soybean abiotic stress responses, and chromatin modifications in response to stressful environments and draws attention to the regulatory potential of circular RNA in post-transcriptional processing.

## Introduction

Crop productivity is affected by a series of abiotic stresses, such as an imbalance in soil nutritional composition, flooding, drought, high salinity, high/low light, and temperature, which adversely affect plant growth and development. Plants overcome environmental variation through gene expression regulation as a mechanism to adjust their physiological functions to new conditions (Soma et al., 2021; Halder et al., 2022). Epigenetic regulation is one of the strategies plants use to achieve stress homeostasis that involves modifications in the chromatin status for modulating gene activation or inactivation at the transcriptional and post-transcriptional levels, and is less explored by researchers, compared to other levels of regulation (Akhter et al., 2021; Miryeganeh, 2021). After being exposed to unfavorable conditions, epigenetic marks are retained and allow plants to cope with a future stressful situation, functioning as a stress memory. Although these modifications do not involve the alteration of bases in the genome, these epigenetic modifications are heritable and prepare the offspring of stress-treated plants to adverse environmental situations (Friedrich et al., 2019; Turgut-Kara et al., 2020). 

Epigenetic regulation includes a complex network of interchangeable components such as histone variants, chromatin remodeling complexes, and non-coding RNAs. Moreover, histone post-translational modifications and DNA methylation, which modify chromatin configuration and DNA accessibility to regulate transcription without altering the coding sequences, have been well documented, and we will explore them in this review (Richards, 2011; Ali et al., 2022; Yung et al., 2022). 

Histones are nuclear proteins that interact with DNA strands and aid the packing of chromatin. Their interaction with DNA occurs mostly due to their basic characteristic, rich in positively charged amino acid residues such as lysine and arginine. Different chemical marks can modify histones at different positions (Zhang et al., 2007; Gates et al., 2017; Demetriadou et al., 2020). The histone lysine residues, mainly present in the N-terminal region, are covalently modified by methylation, acetylation, phosphorylation, and ubiquitination. These modifications alter the activity of the genes involved in the core histones. More than one histone mark generally co-exists at a single histone tail or nucleosome (Ruthenburg et al., 2007; Zhao et al., 2018).

The addition and removal of these histone marks are catalyzed by specific enzyme complexes conserved in angiosperms. They include histone acetyltransferases (HATs), histone deacetylases (HDACs), histone methyl-transferases (HMTs), and histone demethylases (HDMs) (Pandey et al., 2002; Panara et al., 2022). The covalent modifications present on histones can be read by specific protein domains and subsequently trigger downstream signaling events (Liu et al., 2018). Histone modifications have been extensively investigated and characterized in plants, and their effects vary depending on the type of modification and which positional residue is modified (Gates et al., 2017).

**Figure 1 -:**
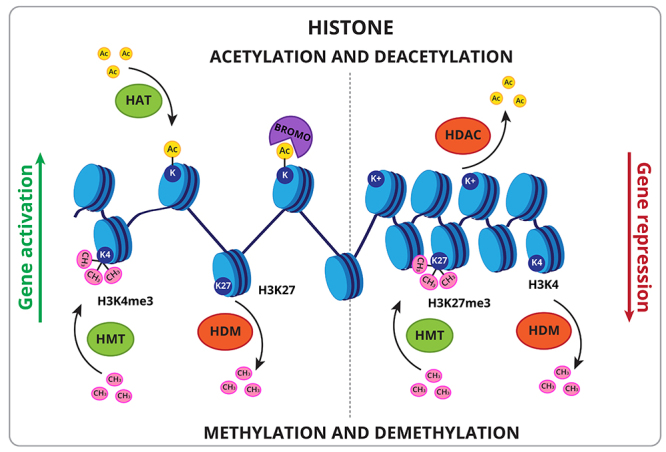
Histone epigenetic marks. Histone modifications associated with gene activation (left) and with gene repression (right). Histone acetylation involves histone acetyltransferase (HAT) that mediates the ligation of acetyl groups (Ac) to lysine residues (K^+^) of histones that form the nucleosome (light-blue circles), and as a result an open configuration of the chromatin. Readers as Bromodomain proteins (BROMO) are needed to mediate downstream biological responses. Histone deacetylation involves histone deacetylases (HDAC) to remove acetyl groups of histones (Ac) increasing their affinity to DNA and a close configuration of the chromatin. Histone methylation and demethylation occur through the activity of histone methyl transferases (HMT) and demethylases (HDM), respectively. Tri-methylation of the fourth lysine of histone 3 (H3K4me3) and demethylation of lysine 27 of histone 3 (H3K27) results in gene activation, whereas trimethylation of lysine 27 of histone 3 (H3K27me3) and demethylation of the fourth lysine of histone 3 (H3K4) results in gene repression.

Acetylation of the ε-NH_3_^+^ residues of lysine present in histone tails neutralizes their positive charge, decreasing their DNA affinity and altering the accessibility of transcription factors to the template DNA chain (Figure 1). As a consequence, histone acetylation tends to induce gene activation (Shahbazian and Grunstein, 2007; Shu et al., 2021). On the other hand, the removal of histone acetylation restores the positive charges of lysine residues and increases their affinity to DNA, triggering gene repression and silencing (Chen and Wu, 2010; Jing et al., 2021). 

The effects of histone methylation vary depending on which residue is modified (Figure 1). Tri-methylation of the fourth lysine of Histone 3 (H3K4me3) accumulated at the transcription-start site activates transcription, while di-methylation in H3K9 (H3K9me2) and tri-methylation in H3K27 (H3K27me3) suppress transcription (Jackson et al., 2004; Hu and Du, 2022). These mechanisms are highly conserved in eukaryotes and are key players in the regulation of gene expression in plants. 

Different from acetylation, histone methylation does not alter the charge of amino acid residues. The presence or absence of methyl groups in the lateral chain of lysine and arginine amino acids alters the association of histones with protein readers, culminating in the remodeling of chromatin structure and activating or repressing gene expression (Liu and Min, 2016; Scheid et al., 2021). 

**Figure 2 -:**
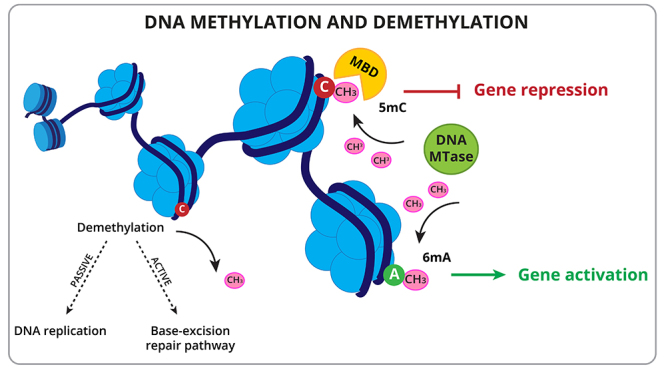
DNA methylation and demethylation. DNA methylation occurs in cytosine (C) and adenine (A) bases mediated by DNA methylase proteins (DNA MTase), and results in gene repression or gene activation, respectively. Methyl binding domain proteins (MBD) have been identified as readers of methylated cytosine to initiate a transcription response. Demethylation occurs through base excision and repair (an active process) or by DNA replication (passive process).

DNA methylation is another epigenetic mark in plants (Figure 2). It consists in the addition of a methyl group to the sixth carbon of the adenine ring (6mA) or the fifth carbon of the cytosine ring (5mC). Cytosine methylation in DNA, in all cytosine sequence contexts, including CG, CHG, and CHH (where H represents A, T, or C), is associated with repression of chromatin in gene promoters and with repression of gene transcription. This modification can be mediated by DNA methyltransferases and non-coding RNAs. In some cases, DNA methylation can also promote gene expression, which has recently been shown to be partially mediated by the DNA methyl-readers SU(VAR)3-9 homologs SUVH1 and SUVH3 (Harris et al., 2018; Xiao et al., 2019).

Methylation on the sixth position of the adenine ring (6mA) has been detected in the genome of Eukaryotes, including plants, such as *Arabidopsis* (Liang et al., 2018a) and rice (Zhou et al., 2018). The potential functions of 6mA include both transcriptional activation and silencing, transgenerational chromatin regulation, and stress responses.

Soybean (*Glycine max* (L.) Merril) is a major crop worldwide because of its protein and oil content, used as a human and animal food source, for biodiesel and fiber production. 

This culture is also important for its ability to improve soil properties through its deep and proliferative root system and its ability to fix atmospheric nitrogen in association with *Bradyrhizobium* bacteria (Pagano and Miransari, 2016). Soybean yields are drastically affected by abiotic stresses (Mutava et al., 2015; Jumrani and Bhatia, 2018), and climate change will strengthen its impact on production, hence a global strategy to minimize crop losses by improving management and plant resilience in response stresses is essential for protecting future food availability (Oerke and Dehne, 2004). To this end, there is an ongoing effort to understand how this species adjusts its metabolism to overcome stressful conditions (Feng et al., 2020; Katam et al., 2020; Kuczyński et al., 2021). This review gathers data about epigenetic and stress memory mechanisms reported for soybean, and another layer of regulation operated by circular RNAs, as a homeostasis mechanism, issues that deserve further investigation by researchers. It intends to unify the latest information on epigenetic marks, factors, and non-coding RNAs that point to candidate genes for toolboxes for soybean breeders to produce new agronomic traits adapted to climate change (Kakoulidou et al., 2021; Liu H et al., 2022). Founded on gaps in the present knowledge, future directions of investigation were also proposed in this review.

## Histone acetylation and deacetylation in response to abiotic stress

### Histone acetylation

HATs and HDAC are the enzymes in charge of histone modifications by acetylation or deacetylation associated with plant responses to abiotic stress (Kim et al., 2015). HATs act in response to drought, salinity, and heat stresses in *Arabidopsis*, Chinese cabbage (*Brassica rapa*), poplar, rice, and tomato (reviewed in (Ueda and Seki, 2020). At least three distinct families of HATs have been characterized: (i) the GNAT (GCN5-related N-terminal acetyltransferases)-MYST family (Neuwald and Landsman, 1997); (ii) the p300/CREB binding protein (CBP) coactivator family (Bannister and Kouzarides, 1996); and (iii) the family related to mammalian TAFII250 (Mizzen et al., 1996). These three families are widespread in eukaryotic genomes. In *Arabidopsis* 12 HAT genes were identified; five from the GNAT/MYST family, five from the CBP family, and two belonging to the TAFII250 family (Pandey et al., 2002). The soybean genome encodes at least 14 HAT, 9 proteins from the GNAT/MYST, three proteins belonging to CBP, and two in the TAFII250 group (Liew et al., 2013). Further studies for soybean HAT characterization and their expression under abiotic stress are needed to understand the mechanisms of stress response by acetylation. 

**Figure 3 -:**
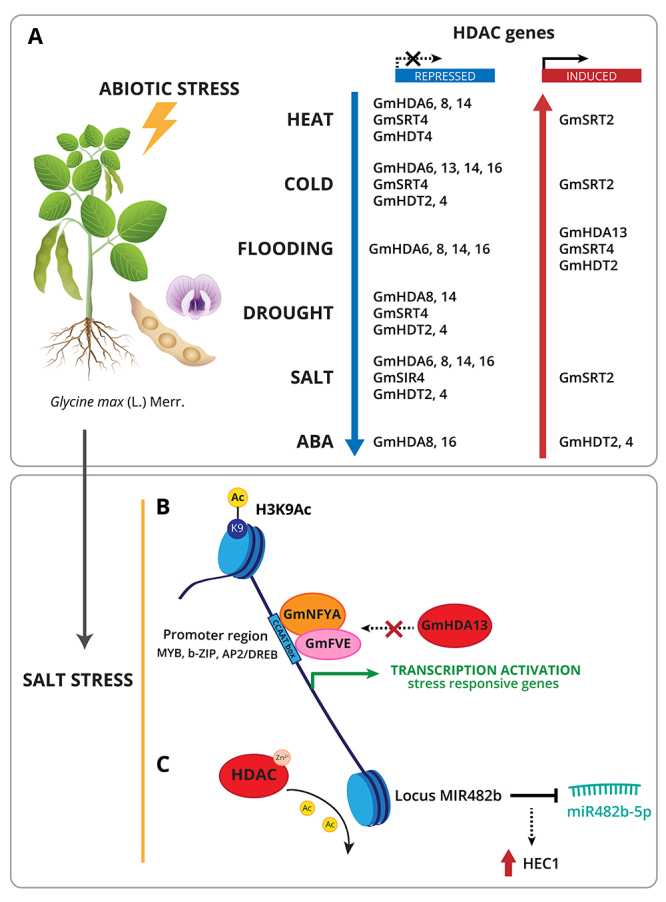
Histone acetylation/deacetylation as epigenetic regulators in soybean under abiotic stress. (A) HDAC gene expression under multiple stresses can be either up or downregulated. Under salt stress: (B) acetylation H3K9Ac has been found in the promoter regions of stress responsive transcription factors (TF), such as MYB, b-ZIP, AP2/DREB. This epigenetic mark was correlated with their differential expression after stress. It has been proposed that GmNFYA forms a complex with GmFVE to bind CCAA box promoters, preventing HDA13 from reaching the promoter and preserving acetylation; (C) MIR482b gene expression is regulated by histone deacetylation, leading to the reduction of its expression and increasing its target gene (HEC1) expression.

In soybean, histone acetylation is an epigenetic mark involved in abiotic stress response (Figure 3B). Song et al. (2012) demonstrated by ChIP analysis, that the activation of transcription factors responsive to salt, such as genes from MYB, b-ZIP, and AP2/DREB families was correlated with an increased level of histone H3K9 acetylation (Song *et al.*, 2012). 

Chilling stress (10 °C) also led to histone acetylation in soybean roots, evaluated by fluorescence signals presence of specific antibodies against H4K12acetyl and H3K9acetyl (Stępiński, 2012). 

Moreover, a report suggested a role for nuclear factor Y subunit GmNFYA in salt tolerance of soybean probably through the regulation of histone acetylation (Figure 3B). That is, under salt stress, GmNFYA likely accumulates and competes with GmHDA13 for interaction with GmFVE, GmNFYA and GmFVE form a complex to bind CCAA box promoters, preventing HDA13 from reaching the promoter and preserving acetylation (Lu et al., 2021).

Recently, Feng et al. (2022) identified a histone acetylation mechanism mediated by SnRK1 kinase in *Glycine soja*. They proposed that during salt stress conditions this protein interacts with the acetyltransferase GsMYST1 for phosphorylation and activation. These proteins are recruited to target genes by the interaction with GsNAC83 transcription factor, forming a heterotrimeric complex. Besides, this complex probably binds to the promoter of the stress responsive COR15B gene and activates its expression by the acetylation of the Histone 4 (K5K8K12K16ace). More studies are needed to assess how conserved this mechanism is in soybean and other species. 

### Histone deacetylation

Studies demonstrated histone deacetylases are involved with ABA and plant stress response (Yang et al., 2018; Ueda and Seki, 2020). In plants, the histone deacetylases (HDACs) can be grouped into three families: (i) the Reduced Potassium Dependency 3 (RPD3)/HDA1, (ii) the Silent Information Regulator 2 (SIR2) and (iii) the histone deacetylases 2 (HD2). HD2 proteins contain a conserved motif (MEFWG) at the amino-terminal region and are zinc-dependent HDACs restricted to plant species (Lee and Cho, 2016). Members of RPD3/HDA1 and the SIR2 families are homologous to yeast HDACs belonging to families with the same names and require Zn^+2^ and nicotine adenine dinucleotide (NAD) as cofactors for deacetylase activity, respectively (Lusser et al., 1997; Pandey et al., 2002; Haigis and Guarente, 2006).

Sixteen HDACs were identified in *Arabidopsis*: ten belong to the RPD3/HDA1 family and are referred to as HDA, four belong to the HD2 family, and were given the name HDT (‘HD-tuins’), and two belong to the SIR2 family and were named SRT (Pandey et al., 2002). 

The soybean (*Glycine max*) genome presents 28 HDAC genes that were identified and characterized based on sequence analysis, chromosomal location, subcellular localizations, tissue and organ-specific expression profile, and stress responsiveness (Yang et al., 2018). Phylogenetic analysis shows that soybean has HDACs that belong to the three families: 18 members of RPD3/HDA1 family, named GmHDA1 to GmHDA18 according to their coordinates on soybean chromosome, four members of SIR2 family with highly conserved Sir2 domains, and six plant-specific HDACs (HD2 family) displaying the conserved amino-terminal conserved motif (Yang *et al.*, 2018). HDAC genes in soybean outnumber *Arabidopsis*, rice, and tomato deacetylase orthologs (Pandey et al., 2002; Fu et al., 2007; Zhao et al., 2014).

Expression analysis under various abiotic stress conditions using quantitative RT-PCR showed that GmHDAC genes were responsive to several abiotic stress treatments (Figure 3A, Table S1). Most of the genes were repressed while few were induced when soybean was exposed to extreme temperatures, flooding, drought, NaCl, ABA treatments (Yang et al., 2018) and nitric oxide (Mirakhorli et al., 2022). Similarly, an RNA-Seq study of soybean under salt stress found three HDAC genes whose expression was modulated (Table S1), being HDAC17, HDT4 and HDT2 repressed (Cadavid et al., 2020b).

HDAC inhibitors, such as suberoylanilide hydroxamic acid (SAHA), have been used to elucidate the relation between histone acetylation and salt stress tolerance. In cassava (*Manihot esculenta* Crantz), roots pretreated with SAHA submitted to high salinity showed induced expression of genes involved in multiple phytohormones biosynthesis pathways, such as abscisic acid (ABA), jasmonic acid (JA), ethylene, and gibberellin. Epigenetic modulation might enhance salt stress tolerance in cassava, consistent with the reduced Na^+^ content and increased K^+^/Na^+^ ratio detected in SAHA-treated plants (Patanun et al., 2016). 

HDAC has also been related to miRNA expression regulation in soybean (Figure 3C). By using SAHA, and high salt treatment, miRNA482bd-5p gene expression is controlled directly or indirectly by an HDAC under salt stress to reduce its transcription with an associated increase in the expression of the target gene HEC1 (Cadavid et al., 2020a).

Reports about acetylation marks in soybean under saline stress evidence community efforts to understand this relevant agriculture problem, but there are still many other stresses critical to be understood. These would be helpful to develop solutions to face agricultural challenges in a climate change scenario. 

## Histones and DNA methylation in response to abiotic stresses

### Histone methylation and demethylation

Histone methylation/demethylation alter gene expression under abiotic stress in plants (Pandey et al., 2016; Kong et al., 2020). HMT and HDM enzymes control this process through the addition/removal of a methyl group to basic residues (Hu and Du, 2022). HMTs methylate Arg and Lys histone residues, namely Arg methyl-transferases (PRMTs) and histone Lys methyl-transferases (HKMTs). Modifications involving histone methylation in *Arabidopsis* contribute to both repression (symmetric H4R3me2, H3K9me2/3, and H3K27me3) and activation marks (asymmetric H4R3me2, H3K4me3, and H3K36me2/3, (Liu et al., 2010; Wang et al., 2016)). *Arabidopsis* HMTs act in response to dehydration, drought, and salinity stresses (reviewed in Ueda and Seki, 2020).

All the known HMTs in plants have a highly conserved domain, SET (Su(var)3-9, Enhancer-of-zeste, Trithorax), which was also named SDG (SET domain groups) proteins (Ng et al., 2007). Target sites for each HKMT and PRMT include: H3K4 (ARABIDOPSIS TRITHORAX [ATX]1/2/3/4/5) methylation; H3K9 ([SUVH]1/2/3/4/5/6/7/8 and [SUVR]1/2/4/5) methylation; H3K27 (ATXR5/6, SWINGER, MEDEA, and CURLY LEAF) methylation; H3K36 (SDG4/8/25/26) methylation; H4K20 (SUVH2) methylation; H3R17 (AtPRMT4a/4b) methylation; and H4R3 (AtPRMT1a/1b/5/10) methylation. In soybean were identified 47 SDG, being 15 PRMTs (Liew et al., 2013).

**Figure 4 -:**
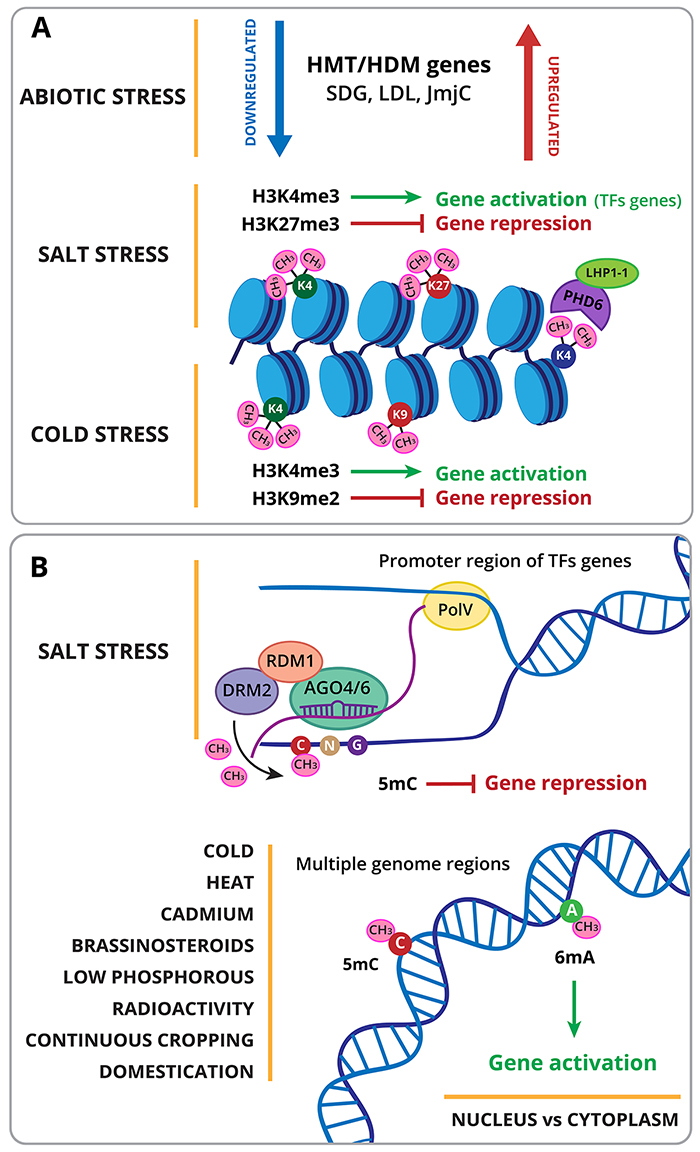
Methylation as epigenetic regulators in soybean under abiotic stress. (A) Histone methyltransferases (HMT) and demethylases (HDM) can be either increased or reduced under stress. Under salt stress: activation of transcription factors (TFs) was correlated with a higher level of histone H3K4me3 and gene inactivation with the H3K27me3. Under cold stress H3K4me3 activation and H3K9me2 repression mark were observed. Plant homeodomain fingers 6 (PHD6) reads low methylated histone H3K4me0/1/2 but not H3K4me3. Upon binding to low methylated histones, the amino-terminal region of PHD6 interacts with its LHP1-1/2 coactivator to form a transcriptional activation complex. (B) Promoter regions of TFs genes are differentially methylated on DNA under salt stress. Differential DNA methylation was observed in soybean under cold, heat, cadmium, brassinosteroids, low-phosphorus, radioactivity, continuous cropping stress and domestication.

Conversely, HDMs are in charge of erasing the histone methylation marks. They are divided into two classes: Lys-specific demethylases (LSD), and hydroxylation by Jumonji C (JmjC) domain-containing proteins (JMJ). Both groups of proteins act in an independent catalytic reaction to facilitate the removal of methyl groups from methylated Lys residues, and some JMJ proteins also function as histone Arg demethylases (Chen et al., 2011; Cho et al., 2012; Liu et al., 2017). Six members genes of LSD-like (LDL) protein family in soybean were identified (Table S1, Figure 4A) and a functional characterization that included gene structure, phylogenetic relationships, three-dimensional structure, expression pattern, genetic diversity, and histone demethylase activity, reported they are modulated under abiotic stress (Liu M *et al.*, 2022). Besides, 24 JmjC domain-containing demethylases were identified for this species during a transcriptome analysis of histone modifiers during floral initiation process (Liew et al., 2013).

The correlation of differentially expressed genes with genomic regions associated with histone methylation (H3K27me3) was examined under salt stress in soybean roots using RNA-Seq and ChIP-Seq data (Figure 4A) (Sun et al., 2019). The trimethylation of histone H3 at lysine residues 27 (H3K27me3) is a hallmark of gene silencing (Zheng and Chen, 2011). Findings strongly correlate the inactivation of genes under salt stress with the *de novo* establishment of H3K27me3 in various parts of the promoter or coding regions lacking H3K27me3 in untreated soybean plants (Sun *et al.*, 2019). Likewise, up-regulated genes were correlated with demethylated regions, suggesting abiotic stress can induce changes in chromatin structure and histone epigenetic marks, which accompanied changes in gene expression. In addition, in the same report, the soybean histone modifiers were identified and the expression level in salt-treated plants of HMT and HDM (Jumonji C) was evaluated (Table S1, Figure 4A). According to protein sequence similarity with *Arabidopsis* HMTs, 43 HMT proteins from soybean were identified (Sun *et al.*, 2019). From CURLY LEAF (CLF), ATX, and SDG genes, nine soybean genes were down-regulated, and two genes were up-regulated (Table S1). JmjC proteins demethylate mono-, di, and trimethylated lysines of histones (Chen *et al.*, 2011). In salt-treated plants, three JmjC proteins were down-regulated and one was upregulated, out of the 21 JmjC proteins whose expression level was analyzed (Table S1). The correlation of salt-related genes activation with histone methylation was also observed (Song et al., 2012). ChIP analysis indicated that the activation of MYB, b-ZIP, and AP2/DREB family genes was correlated with an increased level of histone H3K4me3 and a decrease in H3K9me2 (Figure 4A) (Song *et al.*, 2012). 

Chilling stress (10°C) in soybean root tips was studied to evaluate H3K9me2, H3K4me3 modifications (Figure 4A) using fluorescence signals of specific epigenetic mark antibodies. They found that at this temperature transcriptionally active and inactive marks were altered, as a response of soybean stress regulation (Stępiński, 2012).

Even though studies have advanced in the identification and characterization of HMT and HDM genes in soybean and other species, and demonstrated the relationship of this mark with different abiotic stress, the precise network of actors and their effects on stress regulation associated to histone methylation are not yet elucidated. 

### DNA methylation

**Figure 5 -:**
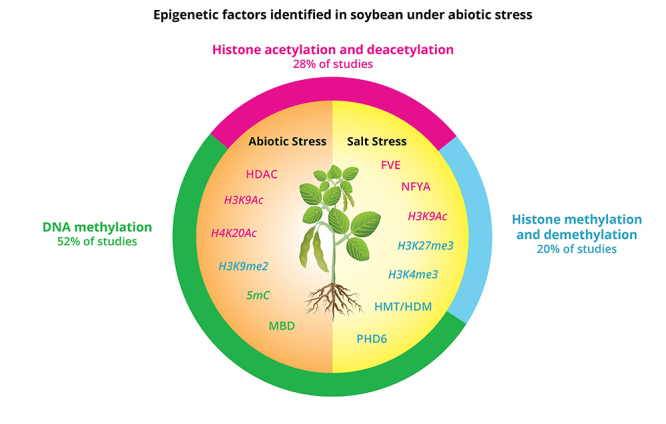
Summary of epigenetic factor identified in soybean under abiotic stress. Epigenetic factors and marks are described in pink for histone acetylation and deacetylation, in blue for histone methylation and demethylation and green for DNA methylation. They are also grouped by salt or other abiotic stresses. The percentages of studies reviewed in each type of modification are presented.

Numerous studies have shown that environmental stress could significantly induce changes in methylation levels in genes accompanied by transcriptional abundance changes (reviewed in Gallego‐Bartolomé, 2020; Akhter et al., 2021). In soybean, DNA methylation have been extensively studied compared to the other marks. In numbers, 52% of the reviewed studies focus on that, while 28% on histone acetylation and 20% on methylation (Figure 5) . In rice, DNA methylation was evaluated under desiccation and salinity stresses by comparing stress-sensitive and tolerant cultivars via bisulfite sequencing. Methylations were positively correlated with the expression of abiotic stress response genes in a cultivar-specific manner (Rajkumar et al., 2020). In tobacco plants, transcriptionally activated genes were found to be hypomethylated under aluminum, salt and low-temperature stress (Choi and Sano, 2007). In *Arabidopsis*, NaCl application caused hypomethylation (Arıkan et al., 2018) and it has been suggested that the salt-induced transcription factor MYB74 is regulated by the RNA-directed DNA methylation (RdDM) in *Arabidopsis* (Xu et al., 2015).

In plants, DNA methylation is found in the CG, CHG, and CHH sequence context (H is either A, T, or C), and it is highly enriched over heterochromatic transposable elements (TEs) and repeats, where it plays a prominent role in their silencing at the transcriptional level. DNA methylation can also trigger gene silencing when it is present in gene regulatory regions (Gallego‐Bartolomé, 2020). Cytosine methylation in plants can be *de novo* established in all contexts by Domain Rearranged Methyltransferase 2 (DRM2) via the RNA-directed DNA methylation (RdDM) pathway (Matzke and Mosher, 2014). RNAs that direct DNA methylation are 24-nt (nucleotide) small interfering RNAs (siRNAs). In addition to siRNAs, longer non-coding RNAs (lncRNAs) specifically referred to as the scaffold RNAs also play a very important role in guiding the methyltransferase to target loci (Zhao and Chen, 2014; Erdmann and Picard, 2020). After biogenesis, small RNAs are loaded into ARGONAUTE 4 (AGO4) and AGO6. The small RNA-AGO complex is recruited to the RdDM target loci by the homologous nascent scaffold RNA through sequence complementarity between the siRNA and the scaffold RNA, and following this interaction, the DRM2 is recruited to the target loci (Figure 4B) (Zhao and Chen, 2014; Zhang H et al., 2018; Erdmann and Picard, 2020).

After DNA replication, multiple DNA methyltransferases are employed to maintain cytosine methylation at different sequence contexts, and CG, CHG, and CHH methylation can be maintained by Methyl-transferase 1 (MET1), Chromomethylase 3 (CMT3), and DRM2 and Chromomethylase 2 (CMT2) enzyme activities, respectively (Finnegan et al., 1998; Stroud et al., 2013; Zhang H et al., 2018). DNA methylation in the symmetric CG and CHG contexts is copied during DNA replication and the nonsymmetrical CHH context is generated *de novo* after DNA replication.

The DNA methylation mark can be removed through DNA demethylation pathways. Both in mammals and plants, the methyl group cannot be directly removed from methylcytosine; instead, the whole methylcytosine base is removed from the DNA backbone and the resulting single-nucleotide gap is filled with an unmethylated cytosine through the base-excision repair pathway in an active way (Figure 2) (reviewed in Liu and Lang, 2020). The demethylation of methylcytosine also occurs in a passive form during DNA replication.

### DNA methylation in response to salt stress

Numerous studies demonstrated that soybean plants respond to abiotic stresses through DNA methylation, possibly as a mechanism to memorized stress. DNA methylation maps were generated in soybean by using bisulfite sequencing under salt conditions. Salt induced alterations of DNA methylation in mRNAs, lincRNAs, and their promoter regions (Chen et al., 2019).

It is well known that both long non-coding RNAs (lncRNAs) and small RNAs can guide DNA methylation or histone modifications by the RdDM pathway. Using transcriptome sequencing of plants submitted to continuous salt stress from seed germination to seedlings, 3,030-induced long intergenic non-coding RNAs (lincRNAs) were identified, as well as their potential functional roles in soybean roots. The main mode of action of lincRNA is regulating neighbor protein-coding genes in *cis* and, therefore, 3,002 nearest genes were identified and proposed as putative targets of lincRNAs in continuous salt stress (Chen et al., 2019).

The induction of DNA methylation by salinity stress in some stress-responsive soybean TFs was previously documented (Figure 4B) (Song et al., 2012). To study the link between cytosine methylation and salt stress response, the expression of GmMYBs, Gmb-ZIPs, GmNACs and GmAP2/DREBs family members was monitored in seedlings exposed to the demethylating agent 5-aza-2’-deoxycytidine (5 ‘ADC) for various periods. As a result, ten TFs genes showed higher expression levels in treated seedlings when compared to mock seedlings (Song *et al.*, 2012). To explore the DNA methylation status of these TF genes, the sequence corresponding to the translation start codon and the promoter region of was subjected to bisulfite sequencing, and results indicated that the Glyma11g02400 (MYB TF), Glyma08g41450 (b-ZIP), Glyma16g27950 (AP2) and Glyma20g30840 promoters were differentially methylated under salinity stress. DNA methylation pattern indicated that methylation affected either CG dinucleotides or CNG/CNN trinucleotides exposed to salt stress (Figure 4B) (Song *et al.*, 2012). These results indicate some TFs genes respond to salinity stress by altering their methylation status. 

### DNA methylation in response to cold, heat, cadmium, sulfur, brassinosteroid and low-phosphorus

Chilling stress affected chromatin configuration of soybean roots (Figure 4B) tip by DNA methylation proved by fluorescence signals of specific antibodies against 5-Methylcytidine (Stępiński, 2012).

Differentially methylated regions (DMRs) in different cytosine DNA contexts were found in response to heat stress in soybean root hairs using whole-genome bisulfite libraries (Figure 4B). The expression analysis of mRNA exhibited some associations between DMRs, genes, and transposons (Hossain et al., 2017).

Cadmium (Cd) stress increased methylation level in a dose-dependent manner in leaf tissues of soybean (Figure 4B), detected by methylation-sensitive amplified polymorphism (MSAP) analysis. From 30 differentially methylated DNA fragments characterized, 15 had sequences that were highly homologous to genes encoding proteins associated with plant stress responses (Sun et al., 2021).

Moreover, it was demonstrated that grafting technology can reduce the total sulfur and Cd content in aboveground parts of soybean, and these traits can be inherited, probably mediated by DNA methylation (Sun et al., 2022). 

To examine if exogenous 24-epibrassinolide (EBR) can improve the salt-alkali resistance, the application of this hormone was studied for alteration of DNA methylation using (MSAP) analysis (Figure 4B). Alteration of levels and patterns of this mark was observed in the whole genome in different tissues (Peng et al., 2021). 

DNA methylation maps were constructed with single-base resolution and genome-wide coverage in two soybean genotypes with different phosphorus efficiencies subjected to low-P and high-P conditions in root tissue (Chu et al., 2020). The DNA methylation levels were slightly higher under Low-P stress in both genotypes (Figure 4B). Integrative methylation and transcription analysis suggested a complex regulatory relationship between DNA methylation and gene expression that may be associated with the type, region, and extent of methylation.

DNA methylation in response to nuclear radiation 

In response to radiation stress, excessive production of ROS can be observed, capable of interrupting different cellular pathways in plants and inducing oxidative damage (Tripathy and Oelmüller, 2012). The adaptation capacity to high concentrations of alkylating and free radical-producing agents is shown as a characteristic feature of Chernobyl plants (Kovalchuk et al., 2004). Likely, hypermethylation is a stress response and general defense mechanism of plants against genome rearrangements (Kovalchuk *et al.*, 2004). 

Soybean (*Glycine max* (L.) Merr. var. Soniachna) was chosen as a model to assess the effect of radioactivity present in Chernobyl environment on plant genome integrity (Figure 4B). For this purpose, the induction and repair of primary DNA damage and the epigenetic contribution to stress adaptation mechanisms were evaluated (Georgieva et al., 2017). An increased level of global genome methylation was observed in plants growing in the Chernobyl area. Soybean plants from the seventh generation of plants grown in radio-contaminated fields exhibit higher methylation levels in CCGG sites in comparison to the control (Georgieva *et al.*, 2017).

### DNA methylation in continuous cropping stress

Long-term continuous cropping imposes limitations to plant growth and compromises soybean quality and yield (Liang et al., 2019). The degradation of soil associated with a decline in soil fertility, disruption of microbial communities, and allelopathic autotoxicity of plants compromise soybean continuous cropping (Ruan et al., 2009; Li *et al.*, 2010; Huang et al., 2013). A genome-wide map of cytosine methylation was generated by bisulfite sequencing and the results were associated with the expression levels of DNA demethylases. Evaluation of stress-tolerant and sensitive cultivars associated the ability to cope with this comprehensive stress with higher DNA demethylation, suggesting it might be a response mechanism in soybean to adjust its metabolism to continuous cropping resistance (Figure 4B). 

### Soybean domestication and genetic improvement have affected the patterns of DNA methylation

Plant domestication shaped plants for the selection of desired traits, along with better growth and performance (Doebley et al., 2006). Compared to wild soybean, cultivated soybean exhibits significant changes in phenotypic characteristics, such as higher biomass, yield (Doebley *et al.*, 2006), and increase in oil content (Zhou et al., 2015). Plant population analyses showed variations in DNA methylation marks among individuals within a species which could result in extensive phenotypic variations (Eichten et al., 2013). Hence, epigenetic variation is an important source of natural variation that might be useful in plant-breeding programs (Gallusci et al., 2017). To better understand the impact of epigenetics on soybean domestication Shen et al. (2018) inspected the variation of DNA methylation by whole-genome sequencing of 45 soybean accessions, including wild soybeans, landraces, and cultivars (Figure 4B). Many DMRs were identified in CG, CHG, CHH contexts across the genome during soybean domestication (wild soybeans versus landraces) and fewer DMRs in the improvement process (landraces versus cultivars). Association analyses between methylation variation and genetic variation in the form of siRNA expression, presence or absence of transposable elements and SNPs revealed that the genetic variation could contribuite to the methylation variations of 22.54% of the total DMRs. The DMRs independent of genetic variation (77.46% of total) occur in regions containing genes related to metabolism that exhibited significant variation in DNA methylation level during the domestication process, especially in genes related to carbohydrate metabolism. 

### DNA methylation in stress-responsive transcription factors

A recent genome-wide analysis of the methylation patterns and differences at CG, CHG, and CHH sites was performed via whole-genome bisulfite sequencing using germinated cotyledons from the soybean curled-cotyledons (cco) mutant and the non-mutant plants (Yang H et al., 2020). The mutant, which has abnormal cotyledons, had more methylated sites but in a slightly lower level than non-mutant plants. Interestingly, genes that were differentially methylated in CHH sites were enriched of TFs, such as GmHDZ20. GmHDZ20 belongs to the HD-Zip I subfamily, which are involved in organ growth, abiotic stress, auxin and light signaling (Ariel et al., 2007). This transcription factor family, and potentially many other TFs, might be regulated by DNA methylation under abiotic stress, which deserves further studies.

### Adenine methylation marks

DNA 6mA modification is a newly discovered epigenetic mark that has been gaining more attention (Liang et al., 2018a). The known effects of 5mC include transposon suppression, gene regulation, and epigenetic memory maintenance (Jones and Takai, 2001; Jones, 2012), but the low abundance of 6mA and the technical limitations of its detection make the study of this epigenetic modification scarce (Ratel et al., 2006). The recent development of third-generation single-molecule sequencing facilitates 6mA detection and allows further studies to unravel unknown effects of this modification (Xiao et al., 2018; van Dijk et al., 2018). 6mA association with gene expression was reported in *Arabidopsis* (Liang *et al.*, 2018b) and rice (Zhang Q et al., 2018) and its levels were positively correlated with the expression of key stress-related genes in rice (Zhang *et al.*, 2018).

A study with wild and cultivated soybean plants found that 6mA sites were extensively distributed across the genome (Yuan et al., 2020). Besides, differences in 6mA modification in cytoplasmic and nuclear DNA for each soybean were investigated at single-nucleotide resolution with SMRT sequencing data (Figure 4B). Nuclear genes with 6mA modification had higher expression than those without modification in both genotypes. As for cytoplasmic gene activity, methylated genes had higher expression in the cultivated soybean than unmethylated genes, but no difference was observed in cytoplasmic genes from wild plants. Hence, it might be interesting to study the relationship between 6mA modification and stress effect in soybean plants to elucidate different mechanisms used for adjustment to environmental variations.

### Histone mark readers

Histone post-translational modifications (PTM) recruit cognate histone binding effector proteins such as histone readers to mediate downstream biological events. The binding of a reader to its cognate histone PTM defines the place and timing of recruitment of the host protein within the genome. Many reader-containing proteins constitute multisubunit enzymatic complexes, in which several readers often with specificities for different PTMs are nearby. Combinatorial readout of the multiple marks by distinct sets of readers provides a lock-and-key mechanism for targeting a particular genomic site that, in turn, is essential for instructing specific biological responses (Andrews et al., 2016).

Chromatin reader domains display distinct binding specificity to different histone PTMs that contribute to the modulation of gene expression in either repressive or active chromatin states. Bromodomain recognizes mainly acetyl-lysine motifs. Plant homeodomain (PHD) fingers are capable of identifying various histone marks, including methylated, unmethylated, and acetylated Lys with different sequence contexts (Figure 4A). Chromodomain family proteins bind preferentially to methylated histone Lys residues. Bromo-adjacent homology (BAH) recognizes distinct histone modifications. Interestingly, many reader proteins contain multiple histone recognition domains that often exist in tandem and function in multivalent chromatin binding to elicit high specificity and avidity to the appropriate epigenetic landscapes (Qian et al., 2018). 

These epigenetic mark readers have been also involved with stress response mechanisms. For instance, finger proteins containing plant homeodomains are involved in various developmental processes and stress responses. In *Arabidopsis*, the PHD finger of SIZ1 (a SUMO E3 ligase) is important for recognizing the histone code and required for SIZ1 function and transcriptional suppression, and abiotic stress response (Miura et al., 2020). In cotton, it is suggested that GhPHDs may act in response to multiple abiotic and phytohormonal stresses (Wu et al., 2021).

The amino-terminal domain of PHD6 from *Glycine max* (GmPHD6) was reported to read low methylated histone H3K4me0/1/2 but not H3K4me3 (Figure 4A). GmPHD6 does not possess transcriptional regulatory ability despite being a DNA-binding protein. Through the PHD finger, GmPHD6 interacts with its LHP1-1/2 coactivator to form a transcriptional activation complex. The overexpression of GmPHD6 using a transgenic hairy root system showed an increased stress tolerance in soybean plants (Wei et al., 2017). In soybean, six Aln1-type PHD proteins were identified in response to ABA, salt, cold, and drought stresses. For instance, the overexpression of GmPHD2 in *Arabidopsis* increases plant tolerance to salt stress (Wei *et al.*, 2009). Those results provide valuable tools for the genetic improvement of soybean. 

DNA methylation marks can be read by a conserved protein family with a methyl-CpG binding domain (MBD) (Figure 2), an important element in the methylation-mediated transcriptional silencing (Grimanelli and Ingouff, 2020). Members of this protein family are capable of recognizing methylated CpG sites and recruiting chromatin remodelers, such as histone deacetylases and histone methyltransferases to repress transcription (Grafi et al., 2007).

The MBD family was first characterized in the *Arabidopsis* genome, which encodes 12 MBD proteins (Zemach and Grafi, 2003). Genome-wide identification and characterization of this family in soybean reported 21 MBD genes, including their gene structure and expression in different tissues, phylogenetic relationship with other MBD plants, and human and protein modeling (Coelho et al., 2022).

## Non-coding RNAs as another layer of gene expression regulation

As previously mentioned in the DNA methylation section, non-coding RNA (ncRNAs) are a diverse group of molecules of different sizes that can act in the regulation of gene expression at the transcriptional level, with methylation being guided by ncRNAs. Even so, small RNAs (miRNAs, tasiRNAs, siRNAs, and tRFs) and long noncoding RNAs (lncRNAs), as well as circular RNAs (circRNAs), can also act as post-transcriptional regulators of gene expression in proteins (Bhogireddy et al., 2021; Li et al., 2021). 

### Soybean microRNAs and abiotic stresses

**Figure 6 -:**
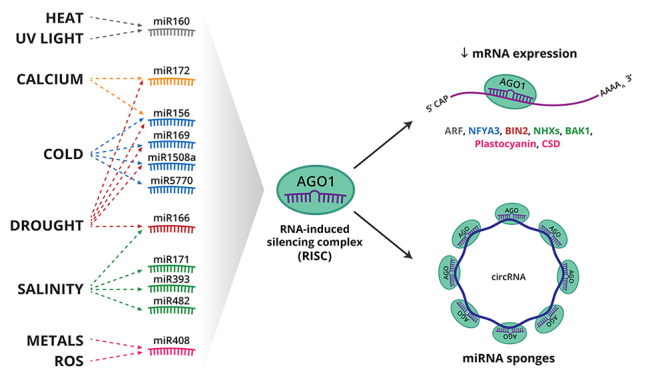
The microRNAs and circRNAs as non-coding RNAs that modulate gene expression in soybean. Different microRNAs have their expression modulated by abiotic stresses and can regulate post-transcriptionally the expression of target genes. Additionally, microRNA can be sponged by circRNAs, molecules that act as repressors of microRNA inhibition.

Several miRNAs have already been associated with the plant responses to abiotic stresses (Figure 6), either by water deficit, saline, metal ions, or nutrient deficiencies such as nitrogen and phosphate (Liu et al., 2008; Kulcheski et al., 2011; Lima et al., 2011; De Lima et al., 2012; Macovei and Tuteja, 2012; De Oliveira et al., 2013; Guzman et al., 2013; Bücker Neto et al., 2015; Kulcheski *et al.*, 2015; Mangrauthia et al., 2017; Millar, 2020; Wang et al., 2021).

Particularly in soybean, a miRNome of stress-responsive microRNAs was described (Ramesh et al., 2019). Besides, an interesting analysis was made correlating the coevolution of MIR genes and their targets along soybean domestication (Liu et al., 2016). It is well documented that miR169 can cleave the soybean transcription factor NFYA3, affecting ABA signaling with a negative effect on water homeostasis, as NFYA3 is implicated in reducing water loss and increasing drought tolerance (Ni et al., 2013).

The miR160 affects pathways associated with auxin-responsive transcription factors (ARF), with impacts both on developmental processes and in response to various environmental factors such as heat, UV, nitrogen availability, and heavy metal concentration (Hao et al., 2022). The miR156, miR169, and miR5770 had similar expression patterns in three soybean varieties in contrast to a cold-sensitive variety, indicating that these miRNAs may play a role in soybean cooling responses (Kuczyński et al., 2021).

An antagonistic effect concerning different abiotic stresses was verified for soybean miR1508a. The overexpression of this microRNA led to a dwarfism phenotype with increased cold tolerance and sugar levels, but with a lower survival rate against water stress (Sun et al., 2020).

In soybean, the NHX (Na^+^/H^+^) antiporter family genes are regulated under saline stress, and are predicted-targets of 75 different microRNAs. Among them, miR393 family targeted GmNHX5 and GmNHX9. Similarly, GmNHX4 and GmNHX6 were the targets of miR166 family members, and miRNA candidates belonging to miR171 family targeted GmNHX1 and GmNHX8 (Joshi et al., 2021). Calcium transport and signaling are also modulated by microRNAs, considering that Ca^+2^ ATPases and channels of small conductance (MSL proteins) are targets for miR156b and miR164 respectively (Zeng et al., 2020). The relative abundance of miR156h, miR172c, and miR166n, and their effects on the epistatic locus Dt2, may explain physiological differences, such as stomatal conductance, and responses to water stress (Zhang et al., 2019).

Comparing two soybean varieties and their responses to saline stress, it was found that there is an increase in the expression levels of BAK1 and BIN2, related to ABA signaling, while miR482 and miR166, their negative regulators, are repressed (Cadavid et al., 2020b). Indeed, 17 miRNAs and 31 putative target genes present an inverse expression pattern in soybean leaves when plants were submitted to salt or osmotic stress (Cadavid *et al.*, 2020a). A link between miRNA regulation and epigenetic regulation was demonstrated in plants treated with the histone deacetylase inhibitor SAHA (suberoylanilide hydroxamic acid), where miR482ab was up-regulated while its target, the HEC1 transcription factor was down-regulated (Cadavid *et al.*, 2020a).

### Soybean circular RNAs and abiotic stresses

Several reviews have described the identification of circRNAs in plants and their correlation to developmental processes, and biotic and abiotic stress (Ye et al., 2015; Wang et al., 2019; Zhao et al., 2019; Wang *et al.*, 2020; Yang X et al., 2020; Zhang et al., 2020; Chand Jha et al., 2021). One of the mechanisms of circRNAs is by acting as sponges of microRNAs associated with argonaut proteins, as reported in *Arabidopsis* (Capelari et al., 2019). Recently the mechanism, well established in animals, was also demonstrated in rice plants by the deletion of multiple circle RNA loci by CRISPR-Cas9, which revealed Os06circ02797 as a putative sponge for OsMIR408 in rice (Zhou et al., 2021).

The pattern of circular RNAs in soybean is highly affected by low phosphate levels. Indeed, more than 70 circRNAs were differentially expressed under phosphate deficiency than are potential sponge targets for more than 570 miRNAs (Lv et al., 2020). Other soybean circRNAs were implicated in the response of plants to low temperatures, where expression analysis demonstrated that circRNA have their levels increased more than the parental genes (where they are transcriped from) under the time course of stress (Wang et al., 2020). 

## Conclusions

Several efforts have been made to understand soybean regulation mechanisms of gene expression under abiotic stress. There is no doubt that epigenetic factors and marks are involved in order to recover plant homeostasis (Figure 5). A correlation between induction of histone acetylation and the activation of transcription factors genes that respond to stress demonstrates the importance of this epigenetic mark to adjust to adverse conditions. GmHDACs have been characterized and their modulation under various stresses have been proved. Moreover, treatment with HDAC inhibitor established a relation between miRNA gene expression regulation under salt stress and histone deacetylation, representing one more epigenetic network component. Even tough soybean HAT genes have been identified, what is missing is a characterization and expression evaluation under stress conditions, to elucidate epigenetic mechanisms by this histone acetylation mark. 

Works about histone methylation have also been reported. Histone modifiers HMT and HDM were identified in soybean and the expression level in salt-treated plants, and other stresses was respectively evaluated. Structural genomic studies allowed to identify histone modifiers. However, more functional studies could clarify the mechanism they used to regulate stress. The correlation of differentially expressed genes with genomic regions associated with histone methylation (H3K27me3 and H3K4me3) was examined under salt stress in soybean. Besides, H3K9me2, H3K4me3 marks were altered under cold stress, which establishes a relation of these marks with stress homeostasis. The homeodomain finger protein PHD6 is a histone methylation reader associated with salt tolerance and abiotic stress response in soybean. In particular, thoses studies focus on saline conditions, which makes it still necessary to understand how histone acetylation and methylation are involved in other types of stresses, essential to find solutions for environmental changes.

DNA methylation is a widely studied epigenetic mark in soybean. Maps on 5mC have been generated in diverse abiotic stresses, revealing the importance of this mark for plant resilience. Moreover, 6mA marks might be an essential component in the plant stress response that it is worth to study in soybean, taking advantage of the new sequencing technologies. DNA methylation readers such as MBD family genes were also identified and characterized in soybean, advancing in the knowledge of the complexity and specificity of the recognition of epigenetic marks, that drives the transcriptional actions that the cell must perform. More research is needed to discover and characterize other components and additional layers of regulatory mechanisms involved in epigenetic regulation in soybean. 

Studies in soybean identified non-coding RNAs involved in DNA methylation pathways and also, ncRNAs have been showed great importance in abiotic stress regulation under the post-transcriptional layers, such as miRNA and circRNAs (Figure 6). 

## Supplementary Material

The following online material is available for this article:

Table S1 **-**
Epigenetic modifiers regulated in soybean under abiotic stress.
