# Supplementary material for: Beyond transcription factors: more regulatory layers affecting soybean gene expression under abiotic stress
Source: Genet Mol Biol. 2023 Jan 23;46(1 Suppl 1):e20220166. doi: 10.1590/1678-4685-GMB-2022-0166 (PMC9881580; doi:10.1590/1678-4685-GMB-2022-0166)
Supplement: Table S1 - [file 1415-4757-GMB-46-1-s1-e20220166-s1.pdf]

Genetics and Molecular Biology

https://doi.org/10.1590/1678-4685-GMB-2022-0166

Supplementary Material to "Beyond transcription factors: more regulatory layers affecting soybean gene expression under abiotic stress"

Table S1 - Epigenetic modifiers regulated in soybean under abiotic stress.

		Down-regulated		Up-regulated		Unchanged		Reference
**Stress**	**Epigenetic modifier**	**Gene**	**Name**	**Gene**				
**Heat**	**Histone deacetylatases**	**Glyma.05 g021400**	GmHDA6	**Glyma.06 g156000**	GmSRT2	**Glyma.12 g086700**	GmHDA 13	Yang et al., 2018
		**Glyma.05 g192600**	GmHDA8			**Glyma.17 g085700**	GmHDA 16	
		**Glyma.12 g188200**	GmHDA14			**Glyma.11 g189500**	GmHDT 2	
		**Glyma.18g076300**	GmSRT4					
		**Glyma.12 g181400**	GmHDT4					
**Cold**		**Glyma.05 g021400**	GmHDA6	**Glyma.06 g156000**	GmSRT2	**Glyma.05 g192600**	GmHDA 8	Yang et al., 2018
		**Glyma.12 g086700**	GmHDA13					
		**Glyma.12 g188200**	GmHDA14					
		**Glyma.17 g085700**	GmHDA16					
		**Glyma.18g076300**	GmSRT4					
		**Glyma.11 g189500**	GmHDT2					
		**Glyma.12 g181400**	GmHDT4					
**Flooding**		**Glyma.05 g021400**	GmHDA6	**Glyma.12 g086700**	GmHDA13	**Glyma.06 g156000**	GmSRT2	Yang et al., 2018
		**Glyma.05 g192600**	GmHDA8	**Glyma.18g076300**	GmSRT4	**Glyma.12 g181400**	GmHDT 4	
		**Glyma.12 g188200**	GmHDA14	**Glyma.11 g189500**	GmHDT2			
		**Glyma.17 g085700**	GmHDA16					
**Drought**		**Glyma.05 g192600**	GmHDA8					Yang et al., 2018
		**Glyma.12 g188200**	GmHDA14					
		**Glyma.18g076300**	GmSRT4					
		**Glyma.11 g189500**	GmHDT2					
		**Glyma.12 g181400**	GmHDT4					
**Salt**		**Glyma.05 g021400**	GmHDA6	**Glyma.06 g156000**	GmSRT2	**Glyma.12 g086700**	GmHDA 13	Yang et al., 2018
		**Glyma.05 g192600**	GmHDA8					
		**Glyma.12 g188200**	GmHDA14					
		**Glyma.17 g085700**	GmHDA16					
		**Glyma.18g076300**	GmSRT4					
		**Glyma.11 g189500**	GmHDT2					
		**Glyma.12 g181400**	GmHDT4					
		**Glyma.17G120900**	GmHDAC17					Cadavid et al. 2020
		**Glyma.12G181400**	GmHDT4					
		**Glyma.11G189500**	GmHDT2					

		Down-regulated		Up-regulated		Unchanged		Reference
**ABA**		**Glyma.05 g192600**	GmHDA8	**Glyma.11 g189500**	GmHDT2	**Glyma.05 g021400**	GmHDA 6	Yang et al., 2018
		**Glyma.17 g085700**	GmHDA16	**Glyma.12 g181400**	GmHDT4	**Glyma.12 g086700**	GmHDA 13	
						**Glyma.12 g188200**	GmHDA 14	
						**Glyma.06 g156000**	GmSRT2	
						**Glyma.18g076 300**	GmSRT4	
**Salt**	**Histone methyltransferases**	**Glyma.17G215200**	ATX2	**Glyma.06G223300**	ASHR3, SDG4|SET domain group 4			Sun et al., 2019
		**Glyma.11G054100**	CLF, ICU1, SDG1, SET1|SET domain-containing protein	**Glyma.16G207200**	ASHH3, SDG7|histone-lysine N-methyltransferase ASHH3			Sun et al., 2019
		**Glyma.13G186800**	SUVH1|SU(VAR)3-9 homolog 1					Sun et al., 2019
		**Glyma.07G056000**	SDG14, ATX3|SET domain protein 14					Sun et al., 2019
		**Glyma.06G151500**	ATXR5, SDG15|ARABIDOPSIS TRITHORAX-RELATED PROTEIN 5					Sun et al., 2019
		**Glyma.01G188000**	CLF, ICU1, SDG1, SET1|SET domain-containing protein					Sun et al., 2019
		**Glyma.15G224400**	SUVH3, SDG19|SU(VAR)3-9 homolog 3					Sun et al., 2019
		**Glyma.20G005400**	SDG22, SUVH9, SET22|SU(VAR)3-9 homolog 9					Sun et al., 2019
		**Glyma.12G196800**	SDG2, ATXR3|SET domain protein 2					Sun et al., 2019
**Salt**	**Histone demethylases**	**Glyma.04G192000**	REF6|relative of early flowering	**Glyma.10G029800**	2-oxoglutarate (2OG) and Fe(II)-dependent oxygenase superfamily protein			Sun et al., 2019
		**Glyma.20G181000**	ELF6|Zinc finger (C2H2 type) family protein/transcription					Sun et al., 2019

		**Down-regulated**		**Up-regulated**		**Unchanged**		**Reference**
			factor jumonji (jmj) family prote					
		**Glyma.20G235300**	Zinc finger, RING-type;Transcription factor jumonji/aspartyl<br>beta-hydroxylas					Sun et al., 2019
**Shade**		**Glyma.09G185800.1**<br>**Glyma.14G216900.1**<br>**Glyma.17G255500.1**	*GmLDL1b*<br>*GmLDL3a*<br>*GmLDL3b*	**Glyma.07G090100.1**<br>**Glyma.06G250100.1**	*GmLDL1a*<br>*GmLDL2*	**Glyma.02G159100.1**	*GmFLD*	Liu et al., 2022
**Heat**				**Glyma.02G159100.1**<br>**Glyma.07G090100.1**<br>**Glyma.09G185800.1**<br>**Glyma.06G250100.1**<br>**Glyma.14G216900.1**<br>**Glyma.17G255500.1**	*GmFLD*<br>*GmLDL1a*<br>*GmLDL1b*<br>*GmLDL2*<br>*GmLDL3a*<br>*GmLDL3b*			Liu et al., 2022
**Cold**				**Glyma.02G159100.1**<br>**Glyma.07G090100.1**<br>**Glyma.09G185800.1**<br>**Glyma.06G250100.1**<br>**Glyma.14G216900.1**<br>**Glyma.17G255500.1**	*GmFLD*<br>*GmLDL1a*<br>*GmLDL1b*<br>*GmLDL2*<br>*GmLDL3a*<br>*GmLDL3b*			Liu et al., 2022
**Drought**		**Glyma.14G216900.1**<br>**Glyma.17G255500.1**	*GmLDL3a*<br>*GmLDL3b*	**Glyma.02G159100.1**<br>**Glyma.07G090100.1**<br>**Glyma.09G185800.1**<br>**Glyma.06G250100.1**	*GmFLD*<br>*GmLDL1a*<br>*GmLDL1b*<br>*GmLDL2*			Liu et al., 2022
**Flooding**		**Glyma.14G216900.1**<br>**Glyma.17G255500.1**	*GmLDL3a*<br>*GmLDL3b*	**Glyma.02G159100.1**<br>**Glyma.07G090100.1**<br>**Glyma.09G185800.1**<br>**Glyma.06G250100.1**	*GmFLD*<br>*GmLDL1a*<br>*GmLDL1b*<br>*GmLDL2*			Liu et al., 2022
**Salt**				**Glyma.02G159100.1**<br>**Glyma.07G090100.1**<br>**Glyma.09G185800.1**<br>**Glyma.06G250100.1**<br>**Glyma.14G216900.1**<br>**Glyma.17G255500.1**	*GmFLD*<br>*GmLDL1a*<br>*GmLDL1b*<br>*GmLDL2*<br>*GmLDL3a*<br>*GmLDL3b*			Liu et al., 2022
**Low Pi**				**Glyma.02G159100.1**<br>**Glyma.07G090100.1**<br>**Glyma.09G185800.1**<br>**Glyma.06G250100.1**	*GmFLD*<br>*GmLDL1a*<br>*GmLDL1b*<br>*GmLDL2*	**Glyma.14G216900.1**<br>**Glyma.17G255500.1**	*GmLDL3a*<br>*GmLDL3b*	Liu et al., 2022

		Down-regulated		Up-regulated		Unchanged		Reference
**Low N**		**Glyma.14G216900.1**<br>**Glyma.17G255500.1**	*GmLDL3a*<br>*GmLDL3b*	**Glyma.02G159100.1**<br>**Glyma.07G090100.1**<br>**Glyma.09G185800.1**<br>**Glyma.06G250100.1**	*GmFLD*<br>*GmLDL1a*<br>*GmLDL1b*<br>*GmLDL2*			Liu et al., 2022
